# Correction

**DOI:** 10.1080/14686996.2024.2408081

**Published:** 2024-10-02

**Article title**: Conductive single-phase SrMoO_3_ epitaxial films synthesized in pure Ar ambience via plasma-assisted radio frequency sputtering

**Authors**: Mouli Roy-Chowdhury, Cong He, Ke Tang, Hiroki Koizumi, Zhenchao Wen, Subhash Thota, Hiroaki Sukegawa, Tadakatsu Ohkubo and Seiji Mitani

**Journal**: *Science and Technology of Advanced Materials*

**Bibliometrics**: VOL. 25, NO. 1, 2378684

**DOI**: https://doi.org/10.1080/14686996.2024.2378684

When this article was first published online it contained a few errors within the text. These have now been corrected.

The errors and the amendments are shown below:

**Table:** 

Section	Error	Correction
***Thin film structural characterizations***	30-nm-thick	30 nm thick
***Thin film synthesis and film surface characterizations***	*T_S_*	*T*_S_
***Electric transport properties***	<20 nm temperature *ρT* dependence of (T)/*ρ*_300K_ increases with decrease in *Ρt* fitted the (*T*)/*ρ*_300K_ *ρ*(*T*)/*ρ*_300*K*_	< 20 nm temperature *T* dependence of *ρ*(T)/*ρ*_300K_ decreases with decrease in *T* fitted the *ρ*(*T*)/*ρ*_300K_ *ρ*(*T*)/*ρ*_300K_
***Equation 1***	_300*K*_	_300K_
***Equation 2***	_300*K*_	_300K_
***Equation 3***	*V_H_* and *R_H_*	*V*_H_ and *R*_H_

